# Monthly Summary

**Published:** 1873-02

**Authors:** 


### MONTHLY SUMMARY.

*Origin of Pus Corpuscles.*—(*Hoffman.*) — It seems now pretty well established that the statement formerly made by Virchow, that the connective tissue is the one source of all inflammatory cells, was too wide, and that pus corpuscles are, in part at least derived from the white blood corpuscles. The next step of course is to the opposite extreme from that of Virchow, namely, to assert that the connective tissue takes no part in the production of pus corpuscles, and this extreme Cohnheim was not slow to reach. The present paper aims at a solution of the question in an experimental manner. The plan of the author's experiments was, to charge the connective tissue of a living animal with vermillion-granules, and then to cause suppuration to be set up ; and now if vermillion were found in the pus corpuscles it would be inferred that the latter arose from the connective tissue, and if not, that the contrary was the case. He found that when vermillion was injected into the vessels of a rabbit, it could be made to collect in considerable quantity within the connective tissue-cells of a given part, by irritating the part. Having thus got the connective tissue charged he excised a portion of the tissue, and so iuduced suppuration, and found that the pus corpuscles contained no vermillion-granules. He concludes, therefore, that the fixed connective tissue corpuscles do not take part in the formation of pus corpuscles, and that as Cohnheim asserts no source of pus corpuscles except the blood has yet been proved.—*Canada Lancet.*

---

*Artificial Eyes.*—A French paper gives a detailed account of the manufacture of false eyes in Paris, from which the curious fact appears that the average sale per week of eyes intended for the human head amounts to four hundred. One of the leading dealers in this article carries on the business in a saloon of great magnificence ; his servant has but one eye, and the effect of any of the eyes wanted by customers is conveniently tried in this servant's head, so that a customer can judge very readily as to the appearance it will produce in his own head. The charge is about ten dollars per eye. For the poor there are second hand visular organs which have been worn for a time and exchanged for new ones ; they are sold at reduced prices, and quantities are sent off to India and the Sandwich Islands.

*A Simple Method of Arresting Epistaxis.*—While resident at the Philadelphia Hospital, I resorted to the following plan of arresting epistaxis, with entire success:

I was called into the medical ward one night to a patient bleeding profusely from the nose, the simple measures usually resorted to—as cold, solution of tannic acid, alum, etc.—having failed to control it. Not having any of the more efficient means usually employed for the arrest of such a hemorrhage, and seeing the dry tannic acid on the table, I remembered the directions given in cases of infantile coryza by Dr. Albert H. Smith, of this city, for softening the hardened secretion in the nostrils. He recommends the introduction of lard upou a small roll of fine linen wrapped like an ordinary lamplighter.

It occurred to me that a similar roll of paper, moistened with water and coated with the dry tannic acid, inserted into the nose, might be of service. I tried it, with immediate success.

I have since found that old linen answers the purpose better than paper applied as above, as it makes a better carrier, being softer, more flexible, and less liable to break down through excess of moisture. I have also found that the powder adheres better if soft lard be used instead of water.

Any powdered styptic may be employed in the same manner. This plan presents the advantages of being always practicable, and of bringing the powder directly in contact with the mucous membrane without danger of wounding it or breaking down the delicate turbinated bones.

I have tried this repeatedly with uniform success, and believe, if it were resorted to, that the disagreeable operation of plugging would seldom be found necessary.—*Med. Times.*

---

*New Method of Arresting Epistaxis.*—Dr. Marin, of Geneva has discovered a new and simple method of arresting hemorhage from the nasal cavity, by applying pressure to the facial artery at a point immediately beneath the ala of the nose where the vessel can be pressed against the superior maxillary bone. In epistaxis, the hemorrhage is usually confined to the anterior third of one of the nasal fossæ, and as pressure upon the nasal artery causes a diminution of the flow of the blood to this cavity, the hemorrhage is arrested almost immediately and this proceeding is therefore recommended as preferable to that of plugging the posterior nares by the aid of Belloc's sound, in attempting which the surgeon is generlly pretty thoroughly smeared with blood, and is not unfrequently bitten. Dr. Marin has had occasion to give numerous trials to this method suggested by him, and has generally found it effective. In two instances where it failed, plugging the posterior nares was attended with like result.—*Boston Med. & Surg. Journal.*

*On Measuring the Chest.*—Dr. Frœhlich, of Dresden, in a recent number of *Virchow's Archiv*, gives for chest measurements the following simple directions, attention to which would insure uniformity of procedure. The person to be examined should stand in an unconstrained position before the physician, breathing with his mouth shut, and should raise both arms, stretching them out horizontally. A tape not broader than 1 Cm. (about $\frac{3}{8}$ of an inch) should be placed round the chest directly under the inferior angles of the scapulæ behind, and the nipple in front, and should then read off, first after the deepest inspiration and then after the deepest expiration, and both data recorded. The author then sums up the results which he has obtained by this method of observation, of which some of the more important are as follows:—The average circumference of the chest measured in 725 healthy men, 20 years of age, was, after deepest inspiration, 89 Cm. (about 35 in.,) and after deepest expiration, 82 Cm. (about 32¼ inches,) the average play of the chest being thus 7 Cm. A circumference of only 75 Cm. (29½ inches) indicates what the author calls an unripe chest, and should exclude the person from military service. A circumference of 750–759 Mm. should, under exceptional circumstances, be considered sufficient for military service; but when it reaches 760 Mm. (30 inches,) if the person is otherwise healthy, then it ought to suffice.—*Med. Reporter.*

---

*Extirpation of the Parotid Gland.*—Dr. J. H. B. M'Clellan reports in the *American Medical Journal* for October, a case of Extirpation of the entire Parotid Gland, for the removal of a fibroid tumor in that regiou, with ligation of the External Carotid Artery and Jugular Vein, and division of Porto Dura Nerve. The subject was a female, aged 32, otherwise healthy, The tumor was enormons, and not only disfigured the patient, but endangered life by pressure. The operation was a protracted one, and ether was used for anæsthesia. Complete recovery ensued, without return of the tumor, a year having elapsed.—*Pacific Med. & Surg. Journal.*

---

*Saliva a Cure for Rheumatism*—A Correspondent writes to the editor of the *Med. Press & Circular* that he has repeatedly cured himself of rheumatism by rubbing the affected part with his own saliva. As saliva is too common to become an article of traffic, the new treatment is not likely to become popular.

---

*Cough Mixture.*—Take hydrochlorate of morphia, one-half grain; glycerine, two fluid ounces. Mix. Dose, a teaspoonful when the cough is troublesome.—*Druggists' Circular.*

*Old Rubber.*—A fortune awaits the happy inventor who shall teach manufacturers to restore old rubber to the condition in which it was before vulcanization, for, with that secret there would practically be *no consumption* of this invaluable article. The thing has been done, and successfully, and we have ourselves seen pieces of vulcanized rubber, possessing great strength and elasticity, which were made entirely from old car-springs; but it has never been accomplished on a large scale, and awaits the enterprise and ingenuity of some new Goodyear to develop it.

Meantime, old rubber has its uses. By a system of steaming and passing between rollers, it is reduced to a semi-plastic state, and in this condition is used in combination with a coarse fabric for heel stiffening, a purpose to which it is admirably adapted, its water-proof qualities being of especial value. There is in a neighboring city a factory devoted entirely to this branch of manufacture, where several hundred tons of old rubber of all kinds are consumed annually.

Old rubber is also largely used to mix with new raw material in the manufacture of all kinds of rubber goods. It serves to give bulk and weight, and, if it does not increase, it certainly does not lessen the strength of the fabric. It may also be mentioned that powdered soapstone, white-lead, terra alba, and other heavy substances enter largely into the composition of almost all rubber goods, the use of which becomes apparent when it is remembered that they are generally sold by weight.—*Am. Artisan.*

---

*The Use of Tobacco.*—Dr. T. L. Wright, says the injury arising from the use of tobacco is to a great extent negative, although as a deleterious agent we will see tobacco exerting sometimes very positive influences. It would be curious, if it were not pitiful, to fully know what sublime schemes, what profound thoughts, what plans, have melted away and vanished through the dreamy imbecility induced by tobacco smoke. Yet, when the enervating influence of the pleasing siren has passed off, the young and the ambitious, or it may be the old and wise, start once more into activity and seem about to realize their cherished and noble ideals, when, once again, and yet again, forever, the soothing influence of tobacco beguiles and deludes, like a silent dream, every faculty of action and every impulse of energy. That people should become addicted to the use of tobacco at all is only explained by the fact that folks often desire to escape from themselves. In our own country, especially, where the excitement of politics is so frequent and so intense, where the weak minded and ignorant, as well as the intellectual, are so frequently whirled into an abyss of passion, there is little reason to be surprised that so many resort to the quieting, pigmy-making properties of tobacco for relief.—*Lancet and Observer.*

